# IDH2 Deficiency Is Critical in Myogenesis and Fatty Acid Metabolism in Mice Skeletal Muscle

**DOI:** 10.3390/ijms21165596

**Published:** 2020-08-05

**Authors:** Jeong Hoon Pan, Jingsi Tang, Young Jun Kim, Jin Hyup Lee, Eui-Cheol Shin, Jiangchao Zhao, Kee-Hong Kim, Kyung A. Hwang, Yan Huang, Jae Kyeom Kim

**Affiliations:** 1 Department of Behavioral Health and Nutrition, University of Delaware, Newark, DE 19716, USA; jhpan@udel.edu; 2 School of Human Environmental Sciences, University of Arkansas, Fayetteville, AR 72701, USA; 3 Department of Animal Science, Division of Agriculture, University of Arkansas, Fayetteville, AR 72701, USA; tangjs@im.ac.cn (J.T.); jzhao77@uark.edu (J.Z.); 4 Department of Food and Biotechnology, Korea University, Sejong 30019, Korea; yk46@korea.ac.kr (Y.J.K.); jinhyuplee@korea.ac.kr (J.H.L.); 5 Department of Food Science, Gyeongnam National University of Science and Technology, Jinju 52725, Korea; eshin@gntech.ac.kr; 6 Department of Food Science, Purdue University, West Lafayette, IN 47897, USA; kim618@purdue.edu; 7 Department of Agrofood Resources, National Institute of Agricultural Sciences, Rural Development Administration, Jeollabuk-do 55365, Korea; kah366@korea.kr

**Keywords:** IDH2 knockout, skeletal muscle, myogenesis, mitochondrial biogenesis, fatty acid oxidation, UCP1

## Abstract

Mitochondrial NADP^+^-dependent isocitrate dehydrogenase (IDH2) catalyzes the oxidative decarboxylation of isocitrate into α-ketoglutarate with concurrent reduction of NADP^+^ to NADPH. However, it is not fully understood how IDH2 is intertwined with muscle development and fatty acid metabolism. Here, we examined the effects of IDH2 knockout (KO) on skeletal muscle energy homeostasis. Calf skeletal muscle samples from 10-week-old male IDH2 KO and wild-type (WT; C57BL/6N) mice were harvested, and the ratio of skeletal muscle weight to body and the ratio of mitochondrial to nucleic DNA were measured. In addition, genes involved in myogenesis, mitochondria biogenesis, adipogenesis, and thermogenesis were compared. Results showed that the ratio of skeletal muscle weight to body weight was lower in IDH2 KO mice than those in WT mice. Of note, a noticeable shift in fiber size distribution was found in IDH2 KO mice. Additionally, there was a trend of a decrease in mitochondrial content in IDH2 KO mice than in WT mice (*p* = 0.09). Further, mRNA expressions for myogenesis and mitochondrial biogenesis were either decreased or showed a trend of decrease in IDH2 KO mice. Moreover, genes for adipogenesis pathway (*Pparg*, *Znf423*, and *Fat1*) were downregulated in IDH2 KO mice. Interestingly, mRNA and protein expression of uncoupling protein 1 (UCP1), a hallmark of thermogenesis, were remarkably increased in IDH2 KO mice. In line with the UCP1 expression, IDH2 KO mice showed higher rectal temperature than WT mice under cold stress. Taken together, IDH2 deficiency may affect myogenesis, possibly due to impairments of muscle generation and abnormal fatty acid oxidation as well as thermogenesis in muscle via upregulation of UCP1.

## 1. Introduction

Skeletal muscle accounts for more than 40% of the body mass and is one of the crucial insulin-responsive organs with abundant mitochondria [1]. It is considered as the most important tissue for glucose and fatty acid utilization in addition to contractile activity during locomotion [2]. In mammal and birds, muscle is also a site of shivering and nonshivering thermogenesis [3]. The heat production in the skeletal muscle is activated during exposure to severe cold environment. The mechanism of shivering is similar to muscle contraction, while nonshivering thermogenesis is regulated by uncoupling protein 1 (UCP1) in the brown adipose tissue, which shares a common embryological origin with muscle [4,5]. A previous study on skeletal muscle UCP1 gene pathway showed the positive effect of UCP1 on whole-body energy expenditure, indicating a close relationship between UCP1 and energy metabolism [6].

The tricarboxylic acid (TCA) cycle is a series of chemical reactions to release energy via oxidation of acetyl-CoA from macronutrients. In this, mitochondrial NADP^+^-dependent isocitrate dehydrogenase (IDH2) catalyzes the oxidative decarboxylation of isocitrate into α-ketoglutarate with concurrent reduction of NADP^+^ to NADPH [7]. Thus, IDH2 not only acts as a metabolic regulatory enzyme in the forward TCA cycle by catalyzing the conversion of α-ketoglutarate but also serves as a major redox regulatory enzyme through the production of NADPH. As such, it is possible to postulate that impaired IDH2 function may contribute to abnormal phenotypes in mice, such as alteration of fatty acid metabolism that is associated with the TCA cycle. We previously reported that IDH2 deficiency is implicated in high fat diet induced obesity [8] and liver steatosis [9]. However, it has not been examined how IDH2 deficiency is intertwined with muscle development and fatty acid metabolism as well as thermogenesis. Therefore, in this study, we utilized an IDH2 knockout (KO) mice model to explore the effects of IDH2 deficiency on muscle homeostasis. 

## 2. Results and Discussion

### 2.1. Genotype and Phenotype of IDH2 KO Mice

**Figure 1:**
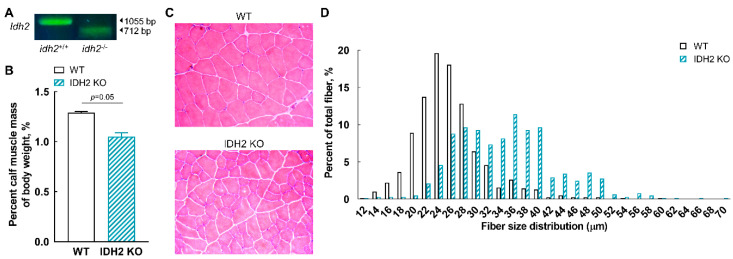
Confirmation of genotype and phenotype of isocitrate dehydrogenase 2 (IDH2) knockout (KO) and wild-type (WT) mice. (**A**) Genotype identification of IDH2 KO mice by conventional PCR analysis. (**B**) Percentage of skeletal muscle mass to body weight of IDH2 KO mice and WT mice (*p* = 0.05). (**C**) Representative images of hematoxylin and eosin staining of mice skeletal muscle (50× magnification). (**D**) Histogram of skeletal muscle fiber size distribution. Muscle mass data are expressed as the mean ± standard error of means and were analyzed by a two-tailed Student’s *t*-test (*n* = 4). *p* < 0.05 was considered statistically significant (SAS version 9.4; SAS Institute, Cary, NC).

To confirm the genotype of IDH2 KO mice, tail DNA genotyping was first carried out. As showed in Figure 1A, the PCR products of IDH2 KO mice were 712 bp, whereas those of wild-type control (WT) mice were 1055 bp, which indicates a successful deletion of IDH2 gene. Then, to assess the phenotypes of the KO mice, we measured the ratio of whole calf muscle mass to body weight for both WT and IDH2 KO mice (Figure 1B). The ratio of calf muscle mass to body weight was significantly decreased in IDH2 KO mice compared with WT (*p* = 0.05). In addition, the morphologies of skeletal muscle tissues were assessed using hematoxylin and eosin (H&E) staining (Figure 1C) and, interestingly, a noticeable shift in fiber size distribution was found in IDH2 KO mice (Figure 1D). The fiber size distribution indicates that IDH2 KO may have an atrophic characteristic of skeletal muscle, which hints that IDH2 deficiency may be linked to myogenesis. These phenotype results prompted us to further examine whether IDH2 deficiency is implicated in myogenesis genes and mitochondria biogenesis genes.

### 2.2. Effects of IDH2 on Myogenesis and Mitochondria Biogenesis

**Figure 2:**
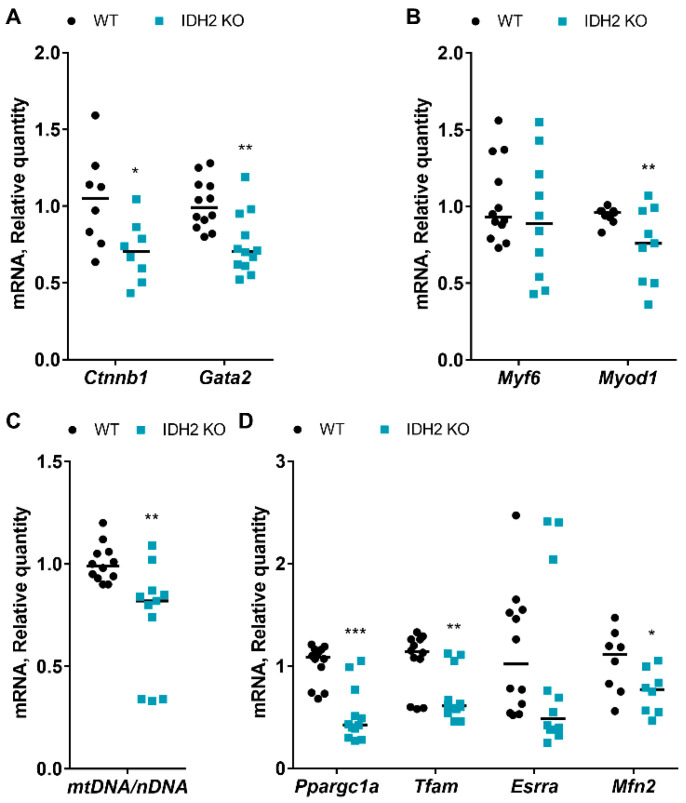
Comparison of mRNA expression of myogenesis and mitochondria biogenesis between IDH2 KO and WT mice. (**A**) mRNA expression of myogenesis genes *Ctnnb1* and *Gata2* in skeletal muscle tissues. (**B**) mRNA expression of specific myogenesis genes *Myf6* and *Myod1* in skeletal muscle tissues. (**C**) The ratio of mitochondria DNA (mtDNA) to nuclear DNA (nDNA) in skeletal muscle tissues. (**D**) mRNA expression of mitochondria biogenesis genes *Ppargc1a*, *Tfam*, *Esrra*, and *Mfn2* in skeletal muscle tissues. All data are expressed as the mean ± standard error of means (*n* = 4; *n* of experimental repeats for qPCR = 8–12). All data were analyzed by a two-tailed Student’s *t*-test. *p* < 0.05 was considered statistically significant (SAS version 9.4; SAS Institute). * *p* < 0.05; ** *p* < 0.01; *** *p* < 0.001.

First, we analyzed beta-catenin (*Ctnnb1*) and GATA-binding factor 2 (*Gata2*) (Figure 2A). We found mRNA expressions were significantly decreased in the IDH2 KO group compared to the WT group. Both *Ctnnb1* and *Gata2* regulate step-specific targets during myogenesis [10,11]. Subsequently, specific myogenesis regulator genes were measured, including myogenic factor 6 (*Myf6*) and myogenic differentiation 1 (*Myod1*). *Myf6* and *Myod1* belong to a family of transcriptional factors that control several skeletal muscle-specific genes [12]. As showed in Figure 2B, *Myod1* was significantly decreased in IDH2 KO mice (*p* < 0.01), indicating that an upstream regulator of myogenesis may be decreased in IDH2-deficient muscle tissues, which is in line with the muscle weight loss as well as atrophic characteristics in IDH2 KO mice.

Then, we examined the ratio of mRNA expression of mitochondria DNA (mtDNA) to nuclear DNA (nDNA) to find out whether IDH2 KO is implicated in mitochondrial biogenesis. The ratio of mtDNA to nDNA is used as an estimate for the mtDNA copy number per cell [13]. We found that the ratio of mtDNA to nDNA was reduced in IDH2 KO group (about 24%) (*p* < 0.01; Figure 2C). In line with the mitochondrial copy number, however, mRNA expression of mitochondria biogenesis genes, peroxisome proliferator-activated receptor gamma coactivator 1-alpha (*Ppargc1a*), mitochondrial transcription factor A (*Tfam*), estrogen-related receptor alpha (*Esrra*), and mitofusin 2 (*Mfn2*) were either decreased or showed a trend of decrease in the IDH2 KO group (Figure 2D). Particularly, the mRNA expressions of *Ppargc1a, Tfam*, and *Mfn2* were significantly decreased in IDH2 KO mice (*p* < 0.001, *p* < 0.01, and *p* < 0.05 respectively; Figure 2D). It is well known that mitochondrial biogenesis (e.g., via *Ppargc1a*, *Tfam*, *Esrra*, and *Mfn2*) plays a major role in muscle development [14,15,16,17]. Interestingly, we found that IDH2 KO muscle had fewer mitochondria copy numbers (approximately 24%), which was in agreement with the expression patterns of mitochondrial biogenesis genes, in particular, *Ppargc1a*, *Tfam*, and *Mfn2*. Hence, it is possible that *Ppargc1a*, *Tfam*, and *Mfn2* may be responsible for reduced mitochondrial contents in the muscle, thereby resulting in a loss of muscle tissue in IDH2 KO mice.

### 2.3. Effects of IDH2 on Adipogenesis, β-Oxidation, and Thermogenesis

**Figure 3:**
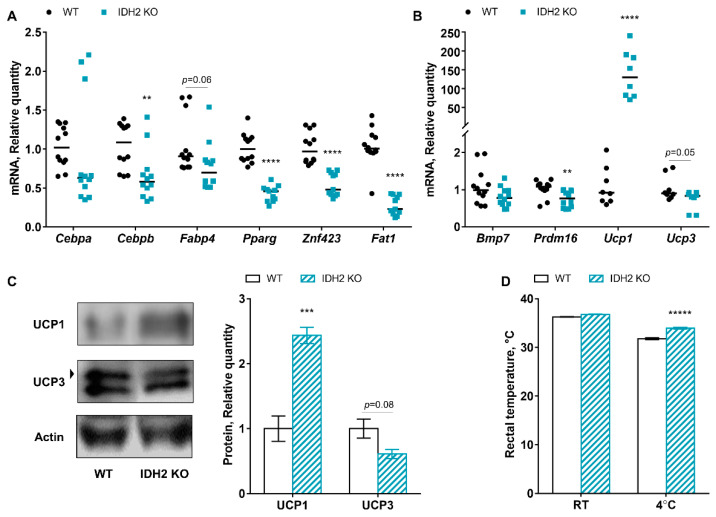
Comparison of mRNA expression of adipogenesis and thermogenesis between IDH2 KO and WT mice. (**A**) mRNA expression of adipogenesis genes, *Cebpa*, *Cebpb*, *Fabp4*, *Pparg*, *Znf423*, and *Fat1* in skeletal muscle tissues. (**B**) mRNA expression of thermogenesis genes, including *Bmp7*, *Prdm16*, *Ucp1*, and *Ucp3* in skeletal muscle tissues. (**C**) Representative western blot images and the relative quantity of proteins for UCP1 and UCP3 proteins are shown as a bar chart. (**D**) Rectal temperatures of mice in response to cold stress at 4 °C. All data are expressed as the mean ± standard error of means (*n* of protein expression and rectal temperature measurements = 4; *n* of experimental repeats for qPCR = 8–12). All data for mRNA expression were analyzed by a two-tailed Student’s t-test. *p* < 0.05 was considered statistically significant (SAS version 9.4; SAS Institute). ** *p* < 0.01; *** *p* < 0.001; **** *p* < 0.0001; ***** *p* < 0.00001.

As aforementioned, IDH2 functions as an important metabolic enzyme in the TCA cycle by catalyzing the conversion of isocitrate to α-ketoglutarate, with concurrent reduction of NADP^+^ to NADPH. Thus, it is very possible that IDH2 deficiency impacts energy metabolism, such as fatty acid metabolism, in muscle tissues. This led us to examine whether IDH2 deficiency influences adipogenesis and β-oxidation. With regard to adipogenesis, the mRNA expression of CCAAT/enhancer-binding protein alpha (*Cebpa*), *Cebpb*, fatty acid binding protein 4 (*Fabp4*), peroxisome proliferator-activated receptor gamma (*Pparg*), and zinc finger protein 423 (*Znf423*) were assessed. We found *Cebpb*, *Pparg*, *Znf423*, and *Fat1* were statistically significantly changed in the IDH2 KO mice, while there was a trend of decrease in *Fabp4* (*p* = 0.06; Figure 3A). However, no difference was noted in β-oxidation (e.g., carnitine palmitoyl transferase I; data not shown). In addition, we analyzed genes related to thermogenesis in muscles, namely, bone morphogenetic protein 7 (*Bmp7*), PR domain containing 16 (*Prdm16*), *Ucp1*, and *Ucp3*. Interestingly, the mRNA expression of *Ucp1* was remarkably increased in IDH2 KO mice (126.62 ± 15.39-fold), although *Prdm16* and *Ucp3* genes were decreased in the IDH2 KO group (Figure 3B).

Due to this stark difference in *Ucp1* between IDH2 KO mice and WT mice, we further examined the protein expression of UCP1 as well as UCP3. We found UCP1 protein expression was also significantly increased in IDH2 KO mice, and there was again a trend of decrease in UCP3 protein expression in IDH2 KO mice, which is consistent with our mRNA expression data (Figure 3C). Further, we carried out a cold stress experiment to test whether the increased UCP1 expression in IDH2 KO mice is linked to body temperature and to confirm whether IDH2 KO mice is resistant to cold stress. As expected, rectal temperature of IDH2 KO mice was higher than that of WT mice under the cold stress condition, strongly indicating that IDH2 deficiency promotes thermogenesis via UCP upregulation. Collectively, our data shows that IDH2 deficiency downregulates muscle adipogenesis, while it upregulates the UCP1 to increase heat in muscle tissues.

In our study, both adipogenesis genes and the thermogenesis gene (i.e., *Ucp1*) in muscle tissues were influenced by IDH2 deficiency in different directions. In particular, *Pparg* and *Znf423* were nearly twice as high in WT mice compared to IDH2 KO mice. These genes are involved in fatty acid synthesis for energy storage [18,19]. *Pparg*, a transcription factor, can induce the expression of fatty acid translocase (protocadherin FAT1 (*Fat1*), also known as cluster of differentiation 36) to modulate fatty acid metabolism in adipose tissues and skeletal muscle tissues [20]. In fact, we found that *Fat1* was significantly downregulated in IDH2 KO muscle (approximately 70% reduction; *p* = 0.0001). Further, *Znf423*, a key initiator of adipogenic differentiation for both subcutaneous and intramuscular fats, is known to enhance the expression of *Pparg* and *Cebpa* [21]. Hence, our results clearly show that IDH2 deficiency might have suppressed adipogenesis, at least in part, via the *Pparg–Fat1* signaling axis in skeletal muscle. A decrease in adipogenic gene expression in IDH2 KO muscle was unexpected as muscle atrophic condition was observed in IDH2 KO mice. Fatty infiltration is one of the common causes of muscle atrophy, accompanied by adipogenesis [22]. Therefore, further studies are warranted to explore factors other than adipogenesis that contribute to the muscle atrophy-like phenotype in IDH2 KO mice. 

On the other hand, UCP1 uncouples the oxidation of fuels, mainly fatty acids, from the production of ATP, and the energy associated with fuel oxidation is released as heat [23]. Given the stark difference in UCP1 expression, our preliminary findings indicate that IDH2 KO may increase significant heat release via oxidation of fatty acids, which is consistent with our previous report. Despite different tissues, in that study, we showed upregulation of UCP1 expression in brown adipose tissues of IDH2 KO mice [8]. The result is also in agreement with data of the current study showing potentially impaired adipogenesis caused by the downregulation of *Pparg* and *Znf423* gene expression. We noticed that, in the IDH2 KO mice liver, fatty acid synthesis related genes (e.g., *Fasn*, *Scd1*) were also downregulated (data not shown, a manuscript is being prepared for separate publication(s)). Together, given the data presented here and previous reports, the increased heat release might have been due to energy expenditure via increased fatty oxidation, which is accompanied by decreased adipogenesis. Further mechanistic studies are clearly needed to understand a causal relationship and to explain implications in muscle atrophy-like phenotype in IDH2 KO mice.

## 3. Materials and Methods

### 3.1. Animal Care

IDH2 KO (*idh2*^−/−^ germ-line KO) mice and their background strain (C57BL/6N) mice were used for the IDH2 KO group and the WT control group (*idh2*^+/+^), respectively. IDH2 KO mice were generated, bred, and maintained as we previously described elsewhere [24]. Both groups (4-week-old; male) were fed with pelleted AIN-76A diet (Central Lab Animals Inc., Seoul, Republic of Korea) and tap water ad libitum for 6 weeks. Temperature (23 ± 2 °C), humidity (50 ± 5%), and a daily 12 h light–dark cycle were maintained in the Central Laboratory Animal Facility of the University of Arkansas. All animal handling and experiments were performed in accordance with protocols approved by the Institutional Animal Care and Use Committee of the University of Arkansas (Protocol Approval Number: 17044).

### 3.2. Genotype Identification and Muscle Tissue Collection

For genetic identification of IDH2 KO mice, tail DNA genotyping was carried out by PCR (Figure 1A). After 6 weeks, all mice were weighted and then killed by exsanguination via cardiac puncture under anesthesia using 2, 2, 2-tribromoethanon (Sigma-Aldrich, St. Louis, MO, USA). Calf skeletal muscle samples (i.e., mixed part of soleus, gastrocnemius, and plantaris muscles) were harvested from both IDH2 KO (*n* = 4) and WT (*n* = 4) mice, and the ratio of skeletal muscle weight to body weight was then measured. All muscle samples were stored in the RNALater solution (Thermofisher Scientific, Waltham, MA, USA) at −80 °C until analyzed.

### 3.3. RNA Isolation and cDNA Synthesis

Stored muscle tissues were retrieved and then homogenized using the Precellys Evolution homogenizer (Bertin Technologies, Rockville, MD, USA). Total RNA was lysed and isolated using the Direct-zol^TM^ RNA MiniPrep Plus Kit in which a genomic DNA elimination reagent (i.e., DNase I) is included to prevent DNA contamination (Zymo Research, Irvine, CA, USA). The quality of isolated RNA was assessed using the conventional A260/280 ratio and A260/230 ratio measurement (SpectraMax i3x; Molecular Devices, Sunnyvale, CA, USA). The total RNA (2 µg) was then reverse transcribed using the High-Capacity cDNA Reverse Transcription Kit (Applied Biosystems, Foster City, CA, USA) following the manufacturer’s protocol. The cDNA samples were stored at −80 °C until analyzed.

### 3.4. Quantitative RT-PCR Analysis

Expression of mRNAs was measured by quantitative real-time RT-PCR analysis using Bio-Rad CFX Connect Real-Time system (Bio-Rad Laboratories, Hercules, CA, USA) in a reaction mixture containing iQ-SYBR Green Supermix (Bio-Rad Laboratories), cDNA, and respective primers. Amplification was conducted under the following conditions: one cycle at 95 °C for 2 min, followed by 40 cycles of denaturation (95 °C for 30 s), annealing (55 °C for 30 s), and extension (72 °C for 40 s). For *Ctnnb1*, *Mfn2*, *Ucp1*, and *Ucp3* expression, primers tagged with TaqMan probe were used in StepOnePlus system (Applied Biosystems). TaqMan Fast Advanced Master Mix was utilized under the following conditions: one cycle at 50 °C for 2 min and 95 °C for 10 min, followed by 40 cycles of denaturation (95 °C for 1 s) and annealing/extension (60 °C for 20 s). Genes of interest were normalized to reference genes (*18S rRNA* or *Actb*). Data were analyzed with Microsoft Excel Software using the ΔΔCT method. All samples were run in triplicates. Detailed primer information (i.e., sequence, assay type, and specific target variant(s)) are provided in Table S1.

### 3.5. Immunoblot Analysis 

Expressions of specific proteins were measured by immunoblot analysis. In brief, total proteins were isolated from muscle tissues and prepared at a concentration of 1 mg/mL by diluting with the Laemmli sample buffer containing 10% sodium dodecyl sulfate and 5% 2-mercaptoethanol. Loaded proteins were separated via sodium dodecyl sulfate polyacrylamide gel electrophoresis and blotted to polyvinylidene fluoride membranes. After blocking the membranes using 5% bovine serum albumin in Tris-buffered saline (0.5 M Tris base, 9% NaCl, and 2% Tween 20; pH 7.8), the membranes were incubated with primary antibodies for 12 h at 4 °C. A complete list of antibodies is available in Table S2. Subsequently, the membranes were washed and then incubated with respective horseradish peroxidase-conjugated secondary antibodies for 1 h at room temperature. Protein bands were visualized using ChemiDoc Imaging System (Bio-Rad Laboratories), and their intensity was quantified using the ImageJ software (National Institutes of Health, Bethesda, MD, USA). The final results were calculated as the ratio of protein/β-actin.

### 3.6. Measurement of Rectal Temperature in Response to Cold Stress

Mice were subjected to cold stress to investigate thermogenic phenotype of IDH2 KO mice. Prior to giving cold stress, rectal temperatures of mice were monitored using an electronic thermistor (Model BAT-12, Physitemp Instruments LLC, Clifton, NJ, USA) equipped with a rectal probe (RET-3, Physitemp Instruments LLC). Subsequently, mice cages were placed in a cold room at 4 °C for 4 h, followed by monitoring rectal temperatures of mice. 

### 3.7. Histological Analysis

Histopathological examinations of H&E staining of calf muscle were carried out in OCT-embedded cross sections. Stained tissue sections were photographed under an upright wide-field microscope (Axioplan 2; Carl Zeiss AG, Oberkochen, Germany) equipped with Axiocam 512 Color Camera (Carl Zeiss AG). Size distribution of muscle fiber was analyzed using the ImageJ software (NIH, Bethesda, MD, USA).

### 3.8. Statistical Analysis

Data are expressed as the mean ± standard error of means and are expressed relative to the WT group except for the percentage calf muscle mass of body weight. Differences between the groups were tested using the two-tailed Student’s *t*-test (SAS Institute, Cary, NC, USA). *p* ≤ 0.05 was considered statistically different.

## 4. Conclusions

In the present study, we found that IDH2 deficiency causes a decrease in muscle mass. To explain this phenotype, we examined myogenesis, mitochondrial contents, and fatty acid metabolism in muscle tissues. Results showed that muscle generation, mitochondria copy number, and adipogenesis were all suppressed in IDH2 KO mice, whereas both *Ucp1* gene and UCP1 protein expressions were remarkably increased. Therefore, for the first time, we report here that IDH2 deficiency may result in the weight loss of muscle tissue, possibly due to impairments of muscle generation and abnormal fatty acid oxidation in muscle. Although our findings provide preliminary evidence to explain muscle phenotype in IDH2 KO mice, additional studies are clearly warranted to explore overall metabolic outcomes of decrease in skeletal muscle mass, especially in the context of fatty acid oxidation and changes in the mitochondrial copy number. Further, muscle type-dependent study is also warranted for in-depth understanding of the role of IDH2 gene in skeletal muscle biology.

## Supplementary Materials

Supplementary materials can be found at https://www.mdpi.com/1422-0067/21/16/5596/s1. Table S1: Primer information, Table S2: Antibody list.

## Abbreviations

18S	18S ribosomal RNA
Actb	beta-actin
Bmp7	bone morphogenetic protein 7
Cebpa	CCAAT/enhancer-binding protein alpha
Cebpb	CCAAT/enhancer-binding protein beta
Ctnnb1	beta-catenin
Esrra	estrogen-related receptor alpha
Fabp4	fatty acid binding protein 4
Fat1	protocadherin FAT 1
Gata2	GATA-binding factor 2
IDH2	mitochondrial NADP+-dependent isocitrate dehydrogenase
KO	knockout
Mfn2	mitofusin 2
mtDNA	mitochondria DNA
Myf6	myogenic factor 6
Myod1	myogenic differentiation 1
nDNA	nuclear DNA
Pparg	peroxisome proliferator-activated receptor gamma
Pppargc1a	proliferator-activated receptor gamma coactivator 1-alpha
Prdm16	PR domain containing 16
Tfam	mitochondrial transcription factor A
Ucp	uncoupling protein, mRNA
UCP	uncoupling protein, protein
WT	wild-type
Znf423	zinc finger protein 423
