# Supplementary material for: IDH2 Deficiency Is Critical in Myogenesis and Fatty Acid Metabolism in Mice Skeletal Muscle
Source: Int J Mol Sci. 2020 Aug 5;21(16):5596. doi: 10.3390/ijms21165596 (PMC7460611; doi:10.3390/ijms21165596)
Supplement: Supplementary file 1 [file ijms-21-05596-s001.pdf]

Supplemental materials

Table S1. Primer information

Primers	Assay type	Forward sequence	Reverse sequence	Target transcript variants
Bmp7	SYBR green	CACCCTCGATACCACCATCG	GCAAGAAGAGGTCCGACTCC	NM 007557.3
Cebpa		GCAAGCCAGGACTAGGAGAT	AATACTAGTACTGCCGGGCC	NM 001287514.1; NM 007678.3
Cebpb		AAGCTGAGCGACGAGTACAAGATG	TTGAACAAGTTCCGCAGGGTGCT	NM 001287738.1
Esrra		GGTGTGGCATCCTGTGAGGC	AGGCACTTGGTGAAGCGGCA	NM 007953.2; XM 006527111.3; XM 030250950.1; XM 017318209.2
Fabp4		CGACAGGAAGGTGAAGAGCATCATA	CATAAACTCTTGTGGAAGTCACGCCT	NM 024406.3
Fat1		CTCCTAGTAGGCGTGGGTCT	CACGGGGTCTCAACCATTCA	NM 001159558.1
Gata2		CCCTATCCCGTGAATCCGC	CCACCTCCATGGTCCACTAC	NM 008090.5
Myf6		GTGGACCCCTACAGCTACAAACC	TGGAAGAAAGGCGCTGAAGAC	NM 008657.3
Myod1		TCTGGAGCCCTCCTGGCACC	CGGGAAGGGGGAGAGTGGGG	NM 010866.2
mtDNA		CGATAAACCCCGCTCTACCT	AGCCCATTTCTTCCCATTTC	AP014941.1
nDNA		CCTTGGGTCCTTGGCTTCGTTCCT	CTCAGCAATCAGCCGTCCAATTCCTA	AC091473.2
Pparg		GATGTCTCACAATGCCATCAG	TCAGCAGACTCTGGGTTCAG	NM 001308354.1; XM 006505743.4; XM 017321455.2; XM 006505739.3; XM 006505738.4; XM

			006505737.4
Ppargc1a	TCCTCTGACCCCAGAGTCAC	CTTGGTTGGCTTTATGAGGAGG	XM 006503779.4; XM 030254205.1; XM 017320718.2; XM 006503776.4; XM 006503774.4
Prdm16	AAGGAGGCCGACTTTGGATG	TTTGATGCAGCTCTCCTGGG	NM 001177995.1; NM 001291026.1; NM 001291029.1; NM 027504.3; XM 006539174.4; XM 006539175.5.4; XM 006539173.4; XM 006539172.4; XM 006539171.4; XM 006539178.4; XM 006539179.1
Tfam	GCTTGGAAAACCAAAAAGAC	CCCAAGACTTCATTTCATT	NM 009360.4; XM 017313918.2
Znf423	GTCACCAGTGCCCAGGAAGAAGAC	AACATCTGGTTGCACAGTTTACACTCAT	NM 001310520.1; NM 033327.2; XM 030243879.1; XM 011248561.3; XM 030243878.1; XM 030243877.1; XM 030243876.1; XM 017313033.2; XM 011248560.3; XM 006531539.3
18S	GTAACCCGTTGAACCCCATT	CCATCCAATCGGTAGTAGCG	NR 003278.3

Primer	Assay type	Assay ID	
Actb		Mm02619580_g1	NM 007393
Ctnnb1		Mm00483039_m1	NM 001165902.1; NM 007614.3
Mfn2	TaqMan probe	Mm00500120_m1	NM 001285920.1; NM 001285921.1; NM 001285922.1; NM 001285923.1; NM 133201.3
Ucp1		Mm01244861_m1	NM 009463.3
Ucp3		Mm01163394_m1	NM 009464.3

Table S2. Antibody list

Antibody	Host/Clonality	Manufacturer (Cat. #)
Anti-β-Actin	Mouse/Monoclonal	Cell Signaling Technology (3700)
Anti-UCP1	Rabbit/Polyclonal	Abcam (ab10983)
Anti-UCP3	Rabbit/Polyclonal	Abcam (ab10985)
